# The Parkin’Play study: protocol of a phase II randomized controlled trial to assess the effects of a health game on cognition in Parkinson’s disease

**DOI:** 10.1186/s12883-016-0731-z

**Published:** 2016-11-03

**Authors:** Sjors C. F. van de Weijer, Annelien A. Duits, Bastiaan R. Bloem, Roy P. Kessels, Jacobus F. A. Jansen, Sebastian Köhler, Gerrit Tissingh, Mark L. Kuijf

**Affiliations:** 1 Department of Neurology, Maastricht University Medical Center, Maastricht UMC+, P.O. Box 5800, 6202 AZ Maastricht, The Netherlands; 2 Department of Psychiatry and Psychology, Maastricht University Medical Center, Maastricht, The Netherlands; 3 Department of Neurology, Radboud University Medical Center, Nijmegen, The Netherlands; 4 Department of Medical Psychology, Radboud University Medical Center, Nijmegen, The Netherlands; 5 Donders Institute for Brain, Cognition and Behaviour, Radboud University, Nijmegen, The Netherlands; 6 Department of Radiology, Maastricht University Medical Center, Maastricht, The Netherlands; 7 School for Mental Health and Neuroscience, Maastricht University, Maastricht, The Netherlands; 8 Department of Neurology, Zuyderland Medical Center, Heerlen, The Netherlands

**Keywords:** Parkinson disease, Mild cognitive impairment, Cognitive functions, Health game, RCT

## Abstract

**Background:**

In Parkinson’s disease (PD), cognitive impairment is an important non-motor symptom heralding the development of dementia. Effective treatments to slow down the rate of cognitive decline in PD patients with mild cognitive impairment are lacking. Here, we describe the design of the Parkin’Play study, which assesses the effects of a cognitive health game intervention on cognition in PD.

**Methods/Design:**

This study is a multicentre, phase-II, open-randomized clinical trial that aims to recruit 222 PD patients with mild cognitive impairment. Eligible patients have PD, Hoehn & Yahr stages I–III, are aged between 40 and 75 years, and have cognitive impairment but no dementia. The intervention group (*n* = 111) will be trained using a web-based health game targeting multiple cognitive domains. The control group (*n* = 111) will be placed on a waiting list. In order to increase compliance the health game adapts to the subjects’ performance, is enjoyable, and can be played at home. From each group, 20 patients will undergo fMRI to test for potential functional brain changes underlying treatment. The primary outcome after 12 weeks of training is cognitive function, as assessed by a standard neuropsychological assessment battery and an online cognitive assessment. The neuropsychological assessment battery covers the following domains: executive function, memory, visual perception, visuoconstruction and language. A compound score for overall cognitive function will be calculated as the mean score of all test Z-scores based on the distribution of scores for both groups taken together. Secondary outcomes at follow-up visits up to 24 weeks include various motor and non-motor symptoms, compliance, and biological endpoints (fMRI).

**Discussion:**

This study aims at evaluating whether a cognitive intervention among PD patients leads to an increased cognitive performance on targeted domains. Strengths of this study are a unique web-based health game intervention, the large sample size, a control group without intervention and innovations designed to increase compliance.

**Trial registration:**

NTR5637 on 7-jan-2016

## Background

Non-motor symptoms in Parkinson’s disease (PD) are now recognized as major contributors to a decreased quality of life [1, 2]. Cognitive impairment is an important non-motor symptom and common in a substantial proportion of PD patients, even in early stages of the disease, and cognitive deficits typically worsen with disease progression [3]. Mild cognitive impairment in Parkinson’s disease (or PD-MCI) is an umbrella term that refers to the heterogeneity of cognitive deficits in multiple domains. It describes the transition from healthy aging to dementia in which cognitive dysfunction is present, but no functional impairment [4]. PD is considered to be a fronto-striatal syndrome that gives rise to cognitive deficits that are particularly apparent when patients need to generate behaviour on the basis of internal rather than external cues, and when they need to flexibly switch between well-learned tasks [5]. Salient cognitive deficits in PD thus usually relate to deficits in attention and executive function, yet the overall cognitive profile is heterogeneous, with co-existing deficits in memory and visuospatial functions also being frequent.

Current treatment strategies for the cognitive deficits are partially effective at best. Even with optimal medical management, cognitive impairment remains a common and incapacitating problem for many PD patients. Therefore, adequate strategies to improve cognitive function and to possibly delay the onset of PD dementia are urgently needed.

The aging brain is thought to retain some degree of plasticity [6], which suggests that older adults may benefit from cognitive training programs. Most studies on the effect of cognitive training programs have been performed in healthy older adults, or in people with cognitive impairment due to vascular pathology or traumatic brain injury. Results showed that these programs could improve multiple domains of cognition [7–9]. So far, only a few cognitive intervention-studies have been conducted in PD patients [10–12]. Some have shown that cognitive training programs improved memory performance [11] and overall cognitive functions [12] after only six weeks of training. Furthermore, cognitive training resulted in a reduced risk of developing PD-MCI at a one-year follow up assessment [12]. Indeed, a recent systematic review by Leung et al. [13] demonstrated a modest effect (g = 0.23, 95 % confidence interval 0.014–0.44, *p* = 0.037) of cognitive training on cognitive function in patients with mild to moderate PD. According to Leung et al. [13], studies in larger samples are needed to examine the abilities of a cognitive training in preventing cognitive decline in PD.

The functional changes in the brain and mechanisms responsible for the associated degeneration process of MCI are unknown [14]. However, several changes in functional connectivity between selective brain regions also take place during this degeneration process. Visualization of these changes helps to localize the responsible underlying mechanisms and may be used as a tool for evaluation of future treatments. In PD-MCI, changes in global patterns of resting-state functional connectivity have been associated with widespread connectivity decrements in several networks, including the default-mode network (DMN) and occipital networks [3, 15, 16]. Most studies demonstrated a pronounced working-memory related under-recruitment of the striatum and dorsolateral prefrontal regions, but also increments of the connectivity of the DMN with posterior cortical regions. The under-recruitment of the striatum may be associated with a reduced capacity for working-memory updating through a decreased phasic release of dopamine [17], but direct evidence linking cognitive changes to underlying brain mechanisms in PD is sparse. A longitudinal study that followed PD patients for three years mainly found functional connectivity changes in the parietal, temporal, and occipital cortices that were associated with cognitive decline [18]. No longitudinal studies have been published on functional network activity changes in PD patients during active cognitive training.

Here, we describe the design of the Parkin’Play study, a multi-centre randomized controlled trial (RCT) that examines the effect of a web-based gaming service (MyCognition AquaSnap) on cognitive function in PD patients with cognitive impairment but no dementia. The gaming service includes both a cognitive training videogame (AquaSnap) and a cognitive assessment (MyCQ™), which respectively trains and assesses the cognitive functions of a player on five core cognitive domains: attention, psychomotor speed, working memory, episodic memory, and executive function. The combination of these two components ensures an intervention adjusted to the (impaired) cognitive performance level of the subjects. Creating a web-based and adaptive health game that incorporates enhanced cognitive training loops may be more engaging, which may result in an increased compliance and effect in PD-MCI. The aim of this study is to determine whether a web-based gaming service designed for cognitive training is a feasible approach, and able to improve cognitive functioning within a three-month time frame in a new cohort of PD-MCI patients. We hypothesize that being able to train at home may optimize compliance to the intervention, as it motivates and rewards the patients.

### Objectives

The primary objective of the Parkin’Play study is to evaluate whether an individually tailored multi-domain cognitive intervention (a health game) leads to an improvement in cognitive performance on various targeted domains, such as executive function, memory, visual perception, visuo-construction, and language. The secondary objective is to study whether the effects persists over time, after the intervention has ceased.

Additionally, given the limited number of studies among PD-patients, it is still unclear whether cognitive improvement due to cognitive training has a neurobiological basis. To provide us with evidence for a mechanistic explanation for the effect of the intervention and to locate the changes in various brain structures, we will assess task-based and resting-state fMRI. Specifically, we will analyse changes in connectivity patterns in the resting-state sensorimotor network, including the supplementary motor area, sensorimotor cortex, and secondary somatosensory cortex [19].

## Methods/Design

### Ethical approval and trial registration

The study is carried out in compliance with the Helsinki Declaration. The local ethics committee of the Maastricht University Medical Centre has approved the study protocol, patient information letter, and the informed consent forms. Informed consent is obtained and signed by the patient prior to the screening session, after the patient is fully informed about the study and the procedures. The Parkin’Play study is registered in the Dutch trial registration under registration number NTR5637.

### Study design

**Fig. 1:**
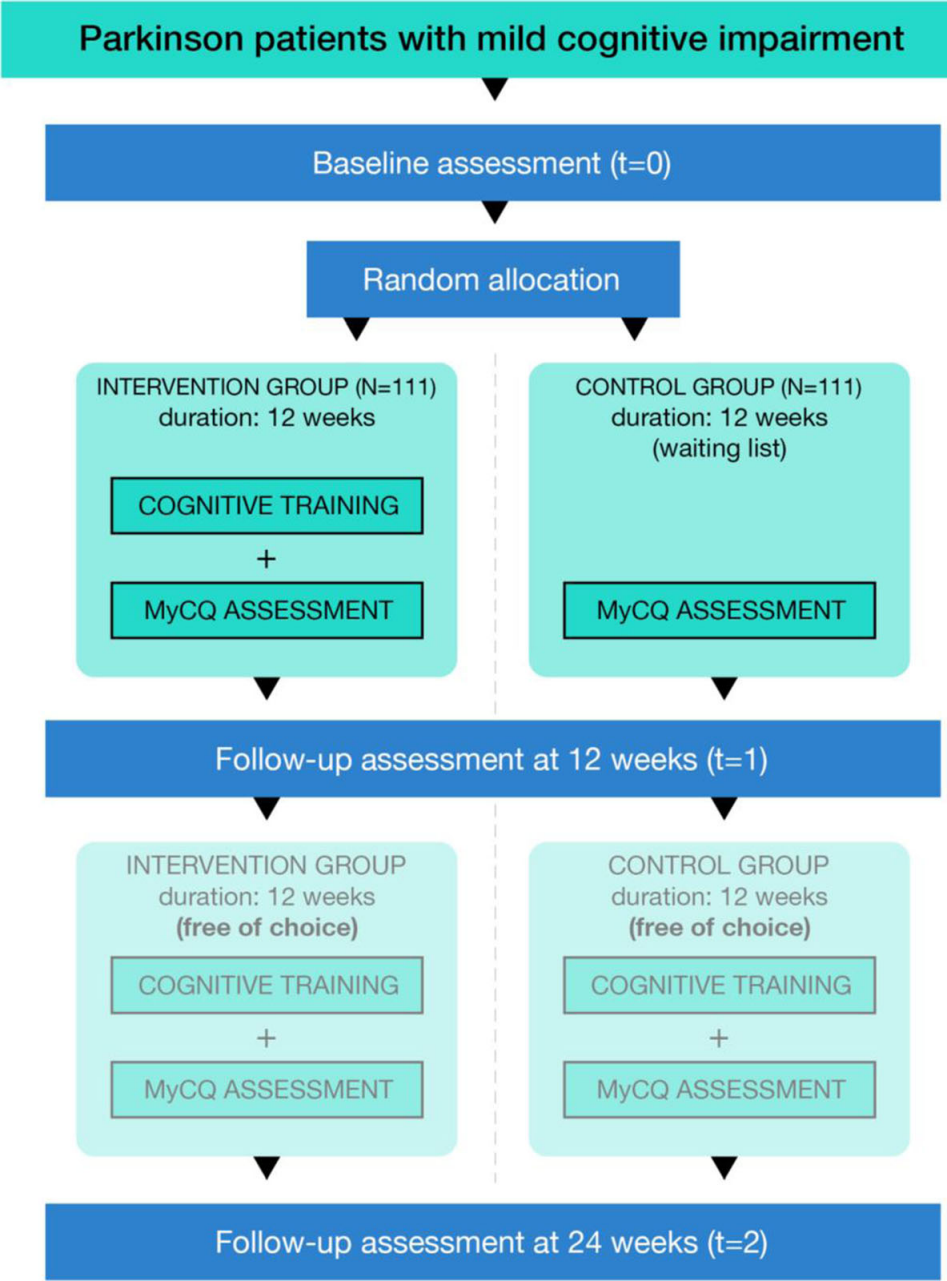
Design of the Parkin'Play study

The Parkin’Play study is a multicenter phase-II open-randomized controlled study that aims to recruit 222 patients with Parkinson’s disease with PD-MCI. Patients are randomly assigned to the intervention (cognitive training) or the control group (waiting list). All participants will undergo three assessments (t = 0, t = 1, and t = 2), which consist of neuropsychological assessments and questionnaires. In 20 patients from each group (total *n* = 40) two fMRI scans will be obtained (t = 0 and t = 1). The duration of the main intervention will be 12 weeks. In order to investigate the compliance and attractiveness of the health game, patients will be given the opportunity to continue playing for an additional 12 weeks (Fig. 1). The study will be analysed based on an intention-to-treat approach and results will be published on behalf of the Parkin’Play investigators.

### Intervention

The total duration of the intervention will be 12 weeks for both groups, with a voluntary extension of the cognitive training for an additional 12 weeks.

#### Cognitive training (intervention group)

The intervention consists of a combination of two software products. To give people insight into their cognitive profile and tailor the intervention towards an individuals’ weaknesses, the participants’ cognitive functions will be assessed using an online assessment tool: MyCognition Quotient (MyCQ™). MyCQ™ is a cognitive assessment tool developed by the company MyCognition. Through ten individual tests, this assessment specifically evaluates the patients’ capabilities in five core cognitive domains: attention, psychomotor speed, working memory, episodic memory, and executive function, consistent with other standard cognitive assessment targeted on Parkinson Disease [20, 21], and with the main neuropsychological domains normally considered as being affected by PD-MCI [22]. MyCQ™ is a 30-min assessment, which provides a personalized cognition score for each individual and a profile of strengths or weaknesses across the five core cognitive domains assessed. The individual tests that comprise the MyCQ™ assessment are based on validated paradigms and they are considered to be revised versions of paradigms that are commonly employed in the assessment of human cognition (see Table 1). The MyCQ™ was compared to the Cambridge Neuropsychological Automated Test Battery (CANTAB) in a population of fifty-five patients with psychiatric disorders [23]. Results indicated that most of the MyCQ™ subtests correlated with the CANTAB subtests of the corresponding domain.

**Table 1:** Individual MyCQ™ tests listed with test equivalents

	MyCQ™ Test	Cognitive domains measures	Paper & pencil test equivalent	Computerised test equivalent
1	Simple Reaction Time	Psychomotor speed	Donders Type A	Detection
2	Choice Reaction Time	Attention	Donders Type B	Identification
3	Go/No Go^a^	Inhibition	Donders Type C	-
4	Verbal recognition memory	Episodic memory	Rey Auditory Verbal Learning Test	CDR Word Recognition
5	Visual recognition memory	Episodic memory	Benton Visual Retention Test	CDR Picture Recognition
6	1-Back^a^	Working memory	-	One Back
7	2-Back^a^	Working memory	-	Two Back
8	Trail Making A	Praxis/psychomotor speed	Trail Making Test Part A	Chase Test
9	Trail Making B^a^	Praxis/psychomotor speed/set shifting	Trail Making Test Part B	Groton Maze Learning Test
10	Coding^a^	Psychomotor speed/attention	Digit Symbol Substitution Test	-

^a^Also indexes elements of executive function

**Fig. 2:**
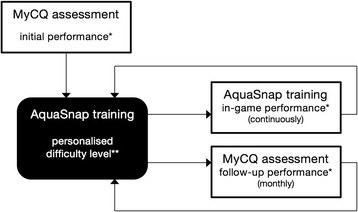
Feedback loops regulating the difficulty and pace of the AquaSnap training

The second product is a custom-made and web-based cognitive training AquaSnap that was built with input from experts of both Radboud University Medical Center and Maastricht University Medical Center. The adaptive cognitive training aims at exercising the cognitive domains of attention, working memory, episodic memory, psychomotor speed and executive function. In AquaSnap, played online on a PC/laptop or Apple iPad, a player is required to explore the ocean in an underwater rover and complete specific tasks by taking pictures of fish. The pictures are worth currency, which can be used to dive deeper into the sea to discover different aquatic environments with rarer fish. The MyCQ™ assessment is carried out monthly and according to an individuals’ profile, AquaSnap adapts the speed and difficulty level of the game. The lower a player’s MyCQ™ score, the more training tasks they need to complete in that specific domain. Each cognitive domain is mainly trained by a particular training loop, while some domains are trained across different tasks (see Table 2). The game develops on different structural levels. At the basic structural level there are the loops, which corresponds to the five first tasks in Table 2. The loops are organized in underwater dives, in which the player undergoes a set of loops. At the Ocean map level, users have to organise their dive in order to both achieve the proposed mission and to discover new areas. The progress of the players on the map, and consequently the growth of difficulty in the training game depend on the coins the players are able to collect during their dives. In this way the game adapts its difficulty to the level of progression reached by the player (Fig. 2). Additionally, as mentioned above, the intensity of the training depends on the individual MyCQ™ scores, as the number of loops for each type of task depends on the score obtained on each cognitive domain. In this way, more impaired domains will receive more intensive training.

**Table 2:** Individual tasks in AquaSnap and trained domains

	AquaSnap task	Description	Cognitive domain
1	Memory shot	Remember the position of the glowing fish in the loop	Working memory
2	Quick shot	Snap the fish as soon as you see it	Processing speed and attention
3	Careful quick shot	Snap the fish as soon as you see it, but be careful not to snap the shocking flash	Attention and executive function (inhibition)
4	Group shot	Snap the group of fish all together	Attention and processing speed
5	Fish Tracker	Remember which fish are glowing after they change position	Working memory
6	Oceanic survey	Remember which fish have you seen at the end of the dive	Episodic memory
7	Missions and map exploration	Achieve the goals proposed by the daily missions and try to discover new areas on the ocean map	Executive function (planning and organizing)

The duration of the cognitive training period is set at 12 weeks with a cumulative duration of 18 h, divided over 36 sessions. In other cognitive training studies, the mean cumulative duration was 17,6 h with a range between 5 and 42 h and the mean training period was 9 weeks with a range between 4 and 24 weeks [10, 11, 24–26].

Time spent per day on gaming will be recorded automatically. There will no pre-set time limits in order to investigate the attractiveness of the games as well as the possibility of testing for addictive behaviour in post-hoc analyses. To prevent attrition due to an inadequate level of difficulty (i.e. too high or too low), coaches will contact the patients every two weeks in the first month to adjust the levels accordingly.

#### Waiting list (control group)

The control group is designed as a waiting list group in order to increase compliance. The participants are required to perform the MyCQ™ assessment monthly. For motivational purposes, the control group is offered to take part in a deferred intervention after 12 weeks (e.g. from week 12 until 24), with the same intensity as the intervention group in the first 12 weeks.

#### Drop-outs and adverse events

Patients who drop out will be encouraged to complete the follow-up measurements. All unwanted and harmful outcomes spontaneously reported by the patients, that may or not be related to the treatment (adverse events), will be recorded according to section 10, subsection 1 of the Dutch Research Involving Human Subjects Act (WMO). Addiction to playing the game will be monitored by recording the amount of playing time and the BIS-11 scoring that measures changes in impulsivity. In case of a serious adverse event, the Ethics committee and relevant authorities (Toetsingonline and principal investigator) will be notified immediately.

#### Medication adjustments

All participants are asked to keep their medication stable during the intervention period. Nonetheless, if medication changes are presumed necessary by the treating neurologist, they are allowed to do so. Any changes in medication will be noted in the case report form.

### Study population

In- and exclusion criteria are shown in Table 3. Patients who are eligible for participating in the study have PD diagnosed by an experienced neurologist, with Hoehn and Yahr stages I–III, and are aged between 40 and 75 years. Furthermore, they have cognitive impairment but no dementia. Neuropsychological assessment including a global measure and a limited battery of standard tests will be used to assess whether MCI is present in accordance with the Level I criteria for the diagnosis of PD-MCI by Litvan et al. [27]. Patients must be on relatively stable dopaminergic medication for at least three months prior to inclusion, or the change in medication does not influence cognition. Patients should not receive any other cognitive therapy during the study period. Also, gamers who play any type of computer games more than one hour per week in the preceding year are excluded from participation.

**Table 3:** In- and exclusion criteria

Inclusion criteria	Exclusion criteria
Idiopathic Parkinson’s disease diagnosed by a neurologist	Medication affecting cognition (such as anticholinergic drugs, benzodiazepine and methylfenidate)
Hoehn & Yahr stage ≤3	Other medical conditions: o Advanced problems in cognitive functioning (MoCA <19/30) o Dementia o Active depression or psychosis and/or treatment with anti-depressant or anti-psychotic drugs o History of active thyroid disease, stroke with residual deficits, severe hypertension or diabetes or head trauma interfering in cognition
Age 40-75 years	Severe auditory of visual deficits
Mild cognitive impairment according to MDS (MCI Level 1 criteria)	No internet at home
Relatively stable dopaminergic medication dose for at least three months prior to the study, or change in medication does not influence cognition. Deemed unlikely to start or stop treatment within the next three months.	fMRI sub-study: o Metal in the body o Claustrophobia

### Recruitment and setting

The treating neurologist will evaluate eligibility of patients using a checklist. After eligible patients have been informed about the study and have agreed to participate, they will be invited for a screening assessment. Informed consent is signed prior to the screening assessment. The inclusion period for the Parkin’Play study will last 18 months. In order to reach target sample size, neurologists from Maastricht University Medical Center, Radboud University Medical Center Nijmegen, and Zuyderland Medical Center Heerlen will be involved in recruitment. Participants are currently being recruited and enrolled. For practical reasons, only patients from Maastricht University Medical Center will be asked to join the fMRI sub-study, prior to the screening assessment. We stop including fMRI participants when the goal of 20 patients per arm is reached.

### Randomization and blinding

After the baseline measurements are performed, participants will be randomly assigned to one of two groups: the Intervention group (IG), or the Control group placed on a waiting list (CG), in a 1:1 ratio. Randomisation (minimisation) will be performed by the Clinical Trial Centre Maastricht (not formally involved in the study or assessments) using the software package ALEA (Formsvision BV). Stratification factors include site location (Maastricht, Nijmegen, or Heerlen) and age group (<60 and > =60 years old). The study is an open-randomised controlled trial, so there will be no blinding for treatment allocation. Nonetheless, all follow-up measurements will be assessed by blinded outcome assessors. Patients will be enrolled by a coordinating investigator, who assigns patients to the intervention groups.

### Outcome measures

The primary outcome is global cognitive function, as assessed by both a standard neuropsychological assessment and the MyCQ™, at baseline and 12 weeks after baseline assessment. The standard neuropsychological assessment consists of various tests covering the following domains: executive function (Stroop Colour Word Test [28], category fluency and letter fluency in parallel versions [29]), memory (Rey Auditory Verbal Learning Test in parallel versions [30], Location Learning Test [31]), visual perception (Judgement of Line Orientation in parallel versions [32]), visuoconstruction (Rey-Osterrieth Complex Figure [33, 34]), and language (Boston Naming Test – Short Form). A compound score for overall (global) cognitive function will be calculated as the mean score of all test Z-scores based on the distribution of scores for both groups taken together. Z-scores of tests with higher scores representing worse performance will be inverted before computing the compound scores. By comparing Z-scores across different neuropsychological tests, the neuropsychological profiles of participants can be directly compared to identify the domains that benefitted most from the intervention.

The participants will also perform a monthly web-based online neuropsychological assessment (MyCQ™), which is part of the intervention program. Since the MyCQ™ is integrated in the AquaSnap health game (i.e., the difficulty level of the game automatically adapts according to MyCQ™ performance), it is not an independent measure of treatment effects, and hence the standard neuropsychological test battery is the primary outcome measure.

### Additional study parameters

#### Non-motor symptoms

Several assessments will be performed at baseline. The Montreal Cognitive Assessment (MoCA) is used to assess global cognition and it is suggested to be more sensitive to cognitive impairment in PD populations in comparison with the Mini Mental State Exam (MMSE) [35]. MoCA includes several neuropsychological items focussing on memory, language, executive function, and visuospatial processing [36]. The Dutch National Adult Reading Task (NART) estimates premorbid intelligence levels. The test is untimed and consists of 50 words with atypical phonemic pronunciation. Each word is presented individually and subjects are asked to read them out loud [37].

Depression will be assessed with the Hamilton Anxiety and Depression Scale (HADS) [38], which has been validated in PD [39]. The Epworth Sleepiness Scale is used to provide a measurement of the subject’s general level of daytime sleepiness [40]. The Cognitive Failure Questionnaire (CFQ) contains 25 questions and provides a self-report evaluation of perception, memory and motor-function in daily life [41]. The Parkinson’s Disease Cognitive Functional Rating Scale (PD-CFR) is a 12-item questionnaire for rating functional abnormalities associated to cognitive impairment in non-demented PD patients [42]. The pre-Rasch-built Overall Disability Scale (pre-R-ODS) can be used to capture functional disability of the patients in a descriptive way [43]. Quality of life will be assessed with the Parkinson Disease Questionnaire (PDQ-39) [44]. The Barratt Impulsiveness Scale 11 (BIS-11) is a 30-item self-report questionnaire that measures impulsive behaviour [45].

#### Motor functioning

The unified Parkinson’s disease rating scale (MDS-UPDRS) part III is used to assess and monitor disability and impairment in PD patients.

#### Compliance

Compliance and attractiveness of the health game will be determined based on the number of dropouts and the cognitive training duration in both groups. Additionally, extended compliance in the intervention group, who continue to play the health game between 12 and 24 weeks from baseline, will be tracked. Comparable studies among both healthy subjects as well as PD patients have found increased cognitive abilities after 6 months follow-up [46, 47] and 1 year follow-up [12]. Based on these studies, we expect to find long term benefits of AquaSnap. Moreover, with a 24 week follow-up measurement, we’re able to test the feasibility of the intervention based on the motivation of the subjects to continue playing AquaSnap.

#### fMRI

MRI imaging will be performed on a 3.0-Tesla unit and in the ON-medication state. Imaging will consist of a neuroradiological protocol (T1-weighted sequences), supplemented with full brain functional imaging (N-back fMRI and resting-state fMRI for functional connectivity imaging). T1 scans will be used to assess macro-structural findings. Volumetric assessment of T1-weighted images will be performed using FreeSurfer software. Task based fMRI will be assessed using SPM and resting state fMRI will be assessed using Melodic. In addition, changes in the resting-state sensorimotor network, including the supplementary motor area, sensorimotor cortex, and secondary somatosensory cortex [19], will be analysed using a robust data-driven approach: independent component analysis (ICA) [48, 49]. Imaging will be performed at the Department of Radiology of the Maastricht University Medical Center. The assessors performing the fMRI baseline and follow-up assessments are blinded for treatment allocation.

### Study activities

Enrolment will take place in the weeks prior to baseline assessment, except for the additional inclusion assessments (see Table 4). The following demographics and general variables are collected at baseline: date of birth, sex, educational level, age at onset, disease duration, and details on Parkinson medication (i.e., drug name, dose, frequency, levodopa equivalence). Details on Parkinson medication will also be collected at both follow-up measurements. Randomization will be performed after the baseline assessment. Primary and additional outcome assessments will be performed at baseline, after 12 and after 24 weeks follow up. The additional fMRI scans will be performed at baseline and after 12 weeks, matching the primary outcome assessments. The intervention group will be required to play the health game from start of the intervention until week 12 for at least 36 sessions of 30 min (three times a week). After week 12, both the intervention as well as the control group will be allowed to continue playing the health game free of choice. All patients will be examined in their on-state.

**Table 4:** Study schedule and assessments

	Timepoint →	-*t*_1_	*t*_0_			*t*_1_	*t*_2_
	Visit # →	0	1			2	3
	Week # →	-1 to -3	0	4	8	12	24
ENROLMENT: Screening & inclusion assessments							
Regular clinical intake		X					
Demographics		X					
Inclusion/exclusion		X					
National Adult Reading Task			X				
Montreal Cognitive Assessment			X				
Epworth Sleepiness			X				
Levodopa equivalent dose (LED)			X			X	X
ASSESSMENTS: Primary outcomes							
Standard neuropsychological battery			X			X	X
MyCQ™ assessment			X	X	X	X	X
Additional outcomes							
Motor function (UPDRS part III)			X			X	X
Self-report questionnaires			X			X	X
Parkinson Disease Questionnaire-39			X			X	X
Pre-Rasch-built Overall Disability Scale			X			X	X
fMRI exclusion criteria		X					
fMRI scan			X			X	
INTERVENTIONS							
Randomization			X				
Game playing (36 sessions of 30 min)			X	X	X	*X*	*X*

Only for subset of patients (*n* = 40)

### Data collection and management

All personnel involved in data collection will review the standard operating procedures (SOP) and manuals. Assessors will be certified in Good Clinical Practice (GCP), certified in performing the MDS-UPDRS-III, and trained in assessing the neuropsychological test battery and other assessments by experienced raters. Data will be collected on paper forms and entered into a web-based data entry portal. Questionnaires are completed digitally and are imported automatically in the electronic database, of which a backup will be made daily. A member of the research team will monitor inclusion progress and data collection progress. After completion of the Parkin’Play study, the database will be approved and locked before data-analysis is set in motion.

### Statistical analysis procedure

Differences between the intervention and control group in global cognitive function (compound score of the neuropsychological test battery) at 12 weeks (primary endpoint) will be compared using independent sample t-tests. In addition, differences between groups in the individual cognitive domains will be tested using multivariate analysis of variance (MANOVA). Change in cognition over time (baseline, 12 weeks, 24 weeks) will be tested using repeated-measures ANOVA. All tests will be two-tailed with alpha set at 0.05. The assumptions of normality and homogeneity of variance will be assessed by inspection of normal probability plots and residual plots. In case assumptions are not met, appropriate data transformations will be used. Secondary outcomes will be presented as means, standard deviations, minimum, maximum, median, lower and upper quartiles. The number of observations and changes from baseline will be presented. Categorical data will be presented in contingency tables as frequencies and percentages. The analyses will be performed on an intention-to-treat basis. A secondary “Per-Protocol” analysis will also be included for the patients who have fully completed the study protocol for the primary endpoint. In order to investigate the effect of ‘missingness’, sensitivity analyses will be performed, including missing data augmentation using multiple imputation by chained equations [45] and maximum likelihood estimation with random effects.

For the main study, interim analysis will be conducted after 40 patients have completed the 12-week cognitive training period, including the corresponding assessments, to find out if the training is particularly beneficial or not. If, after interim analysis, it can be concluded that the training is particularly harmful, we may end the study and analyze the study data. This will only be decided in consultation with the local ethics committee.

### Power and sample size estimate

The Parkin’Play study is powered to show a moderate effect of the AquaSnap cognitive training on the standard neuropsychological assessment. A study in healthy older participants compared a brain training program (Brain Age) to a control condition (Tetris gaming) and found an ANCOVA-based effect size of Cohen’s f = 0.39 (eta^2^ = 0.13) on the Trail Making Test, which assesses cognitive flexibility, often reduced in PD [9]. This reflects a moderate-to-large effect size (equivalent of Cohen’s d of 0.78). The present population includes participants aged between 40 and 75 years old, but with PD. Still, we expect a moderate effect size (0.4–0.5). Therefore, the estimated sample size for detecting a difference between the means of the treatment groups on the compound score of standard neuropsychological tests, with a power of 0.8 and a Cohen’s d = 0.4, at an alpha-level of 0.05, for a two-sided test, and an allocation ratio of 1:1 is 200 patients.

Based on previous cognition training and video game clinical trials the attrition rate is estimated at 10 %, which yields an additional 22 patients that should be recruited for participation [9, 11, 50]. This leaves a total of 222 patients that should be recruited.

## Discussion

The overall aim of this phase-2 multi-center open-randomized controlled clinical trial among PD patients is to evaluate whether a web-based health game designed to train cognition leads to an improvement in cognitive performance on targeted cognitive domains, relative to a control group on a waiting list. The strengths of the Parkin’Play study are the large sample size (*n* = 222), the gamified and home-based training approach, the individually tailored and adaptive training, and the application of fMRI assessments in order to explore underlying brain mechanisms. The addition of the 24-week follow up assessment provides valuable information about maintenance of benefit and about the feasibility of implementing health games in a PD population.

Now that the new MDS-criteria for PD-MCI have been introduced, this study will for the first time study a large sample of patients fulfilling the proposed criteria in a clinical trial [13]. Previous cognitive clinical trials for PD have incorporated a range of cognitive and functional outcome measures, partly based on scales derived from Alzheimer’s disease [51, 52]. This illustrates the lack of consensus on cognitive outcome measures. Furthermore, regulatory agencies may require measures that take into account a functional benefit for the patient. In this study, a compound score from a neuropsychological test battery will be used and complemented with the MyCQ™. The recently validated PD-CFRS scale was chosen to study functional benefit with demonstrated responsiveness over time [53].

The Parkin’Play intervention consists of various innovations. Firstly, performing cognitive training at home is likely to optimize compliance, since PD patients’ reduced mobility might prevent them from attending outdoor activities [12, 47]. Secondly, a task that is too hard may result in anxiety and therefore lead to resistance of collaboration to the treatment (e.g. stop playing the health game). The health game and MyCQ™ assessment combination adapts to the subject’s capabilities and thereby results in a challenging game specifically tailored to the individual. Thirdly, the health game aims at being fun to play, introducing a multi-layer game story opposed to abstract repetitive cognitive brain trainings. These innovations are aimed at increasing the compliance of the intervention and prevent substantial attrition rates associated with study failures.

Cognitive training is thought to have beneficial effects on cognitionthrough activating mechanisms of the brain plasticity. Brain plasticity refers to the capacity of the central nervous system to change or to adapt its structure and function over a lifetime [54, 55]. There is evidence that training on demanding tasks (adaptive training) decreases activation in frontal, parietal, and occipital regions of older adults, which may reflect improved neural efficiency and reduced use of resources [56]. Cognitive training has proved to be able to produce improvement in the main neuropsychological domains involved in PD as memory, attention, processing speed and executive function. Specific studies on the effects of cognitive training on PD have recently been conducted [26]. The results of randomized control trials show that cognitive and affective functions can be improved by cognitive trainings in PD patients. To our knowledge, no cognitive videogame training interventions have been conducted in PD-MDI.

Mild cognitive impairment in PD is generally viewed as a pre-dementia stage in PD, but the functional changes in the brain and mechanisms responsible for the associated degeneration process are unknown. Since the aged brain still retains neuroplasticity [6], it may restore or prevent functional changes of brain network activity. Indeed, in a non-imaging study, Petrelli et al. [12] have found that patients in the cognitive training intervention group had a reduced risk of developing MCI after 1 year follow up. Nonetheless, no longitudinal studies have been published on functional changes in PD patients during cognitive training. With the use of fMRI-imaging, we hope to have a better understanding on functional network activity changes in PD patients as a result of active cognitive training. Visualization of these changes helps to localize the responsible underlying mechanisms and may be used as a tool for evaluation of future treatments.

The Parkin’Play study contributes to a better understanding of cognitive impairment in PD and evaluates a new possible non-pharmacological intervention for PD-MCI.

## Abbreviations

PD: Parkinson’s disease
MCI: Mild cognitive impairment
PD-MCI: Parkinson’s disease mild cognitive impairment
IG: Intervention group
CG: Control group
RCT: Randomized controlled trial
SOP: Standard operating procedures
GCP: Good clinical practice
MDS-UPDRS: Unified Parkinson’s disease rating scale
DMN: Default-mode network
MyCQ™: MyCognition quotient
WMO: Dutch research involving human subjects act
